# Validity and Responsiveness of Preference-Based Quality-of-Life Measures in Informal Carers: A Comparison of 5 Measures Across 4 Conditions

**DOI:** 10.1016/j.jval.2020.01.015

**Published:** 2020-06

**Authors:** Carol McLoughlin, Ilias Goranitis, Hareth Al-Janabi

**Affiliations:** 1 Health Economics Unit, Institute of Applied Health Research, University of Birmingham, Birmingham, England, UK; 2 Centre for Health Policy, Melbourne School of Population and Global Health, The University of Melbourne, Melbourne, Australia

**Keywords:** construct validity, dementia, informal care, mental health, outcome measurement, quality of life, rheumatoid arthritis, responsiveness, stroke

## Abstract

**Objectives:**

Carer quality-of-life (QoL) effects are recommended for inclusion in economic evaluations, but little is known about the relative performance of different types of QoL measures with carers. This study evaluated the validity and responsiveness of 3 care-related QoL measures (the Carer Experience Scale [CES], CarerQoL-7D, and ASCOT-Carer), 1 health-related QoL measure (the EQ-5D-5L), and 1 generic QoL measure (the ICECAP-A).

**Methods:**

Validity and responsiveness were assessed in a UK sample of informal carers of adults with dementia, stroke, mental illness, or rheumatoid arthritis. A questionnaire containing the 5 QoL measures was posted to carers identified through the Family Resources Survey (N = 1004). Hypotheses regarding the anticipated associations between constructs related to the QoL of carers were tested to investigate construct validity and responsiveness.

**Results:**

Each measure exhibited some level of construct validity. In general, larger effect sizes and stronger associations were detected for the ASCOT-Carer and ICECAP-A measures in the pooled sample and across all conditions. The 5 measures did not exhibit clear responsiveness to changes over a 12-month period in care recipient health status or hours of care provided per week.

**Conclusion:**

The results of this study provide initial evidence of the validity of care-related, health-related, and generic QoL (capability) measures in informal carers of adults with 4 highly prevalent conditions. Care-related measures were not always more sensitive to constructs associated with QoL of carers compared with generic measures. The performance of the ICECAP-A was comparable with that of the best-performing care-related measure, the ASCOT-Carer.

## Introduction

Patient health and treatment can be associated with significant spillover effects on the quality of life (QoL) and resource use of informal carers.^1^, ^2^, ^3^, ^4^ Where carer spillovers are included in economic evaluation, the focus is typically on resource use in terms of lost time as a result of caring.^5^^,^^6^ Nevertheless, the importance of including carer QoL effects has been emphasized by the second US panel on cost-effectiveness^7^ and in the guidelines for economic evaluation produced by the National Institute for Health and Care Excellence (NICE) in the United Kingdom.^8^ In practice, economic evaluations rarely consider carer QoL effects.^4^, ^5^, ^6^^,^^9^, ^10^, ^11^, ^12^ The uncertainty over the appropriateness of different QoL measures for carers is commonly cited as one of the main reasons for the exclusion of carer effects.^13^, ^14^, ^15^ For this reason, we focus on evaluating the performance of QoL measures for informal carers in this article.

Carer QoL effects can be measured using standard health-related QoL (HRQoL) instruments, such as the EQ-5D-5L.^16^, ^17^, ^18^, ^19^ Nevertheless, providing care can have a variety of positive and negative effects on all aspects of a carer’s life, including but not limited to, their general health.^20^, ^21^, ^22^ Considering QoL outcomes, such as “relationships” or “feeling supported,” may be more appropriate as they may map onto the issues important to carers better than HRQoL measures.^15^ Care-related QoL (CRQoL) measures, such as the Carer Experience Scale (CES),^23^ CarerQoL-7D,^24^ and Adult Social Care Outcomes Toolkit for Carers (ASCOT-Carer) (which measures social CRQoL)^25^ are designed for use in economic evaluation with the intention of providing additional information to standard methods. Generic QoL measures, such as those capturing well-being and capability, are an alternative to HRQoL or CRQoL measures. The ICEpop CAPability measure for Adults (ICECAP-A) capability measure is in principle capable of picking up care-related and HRQoL issues.^26^

Although there is some promising initial evidence of measure validity for the CES,^27^ CarerQoL-7D,^28^, ^29^, ^30^, ^31^ and ASCOT-Carer,^25^ no evidence has been generated yet for their validity relative to one another and across common diseases. Similarly, evidence on the psychometric properties of preference-based health or generic QoL measures with carers is limited.^19^^,^^32^^,^^33^ To our knowledge, this is the first study to investigate the construct validity and responsiveness of conceptually different QoL measures with informal carers.

## Methods

This study focused on examining the performance of 5 QoL measures: the CES,^23^ CarerQoL-7D,^24^ ASCOT-Carer,^25^ EQ-5D-5L,^34^ and ICECAP-A^26^ in informal carers of adults with dementia, stroke, mental illness, and rheumatoid arthritis. These are high prevalence conditions associated with diverse impacts on carer’s lives. Dementia is a degenerative condition associated with long care hours and physically demanding caregiving^35^, ^36^, ^37^. Mental illness is generally chronic with acute episodes that can require hospitalisation^22^. For mental illness, the questionnaire focused on nonorganic mental disorders (most commonly anxiety disorders, delusional disorders such as schizophrenia, and mood disorders such as depression). Stroke and rheumatoid arthritis often involve younger carers, and the strain of caregiving is often related to the severity of the condition.^38^ As detailed later, the study involved the collection and analysis of QoL data from informal carers via a self-completion postal survey sent to a sample of UK informal carers at 2 time points.

### Sample Frame

NatCen Social Research was commissioned to draw a sample of informal carers from across the United Kingdom (excluding Northern Ireland) to take part in this study from the Family Resources Survey (FRS). The FRS is a continuous household survey that collects information on the income and circumstances of a representative sample of private households in the United Kingdom.^39^ The sample of carers was drawn across 3 waves (2013-2014, 2014-2015, and 2015-2016). All carers were contacted by telephone if they were older than 18 years, cared for a person older than 18 years, and had agreed to be recontacted in the future for research purposes. This resulted in a sample frame of 1004 participants who were willing and eligible to complete the postal survey.

### Survey Development and Data Collection

A survey was developed to capture responses to the QoL measures and constructs related to carer QoL. The individual items of the 5 measures included in the postal survey are presented and conceptually mapped across the 12 domains in Table 1. The conceptual mapping provides a structured framework against which the different items of the QoL measures can be assessed. The CES, CarerQoL-7D, and ASCOT-Carer offer preference-based scores of CRQoL, and each measure is designed for use in economic evaluations. The CES contains 6 items that capture conceptual attributes of the caring experience. Relative weights attached to each of the 6 care dimensions are aggregated to provide a preference-based overall score of caring experiences (0 = bottom state, 100 = top state).^23^ The CarerQoL-7D instrument contains 7 dimensions that can be aggregated and weighted by their severity with a tariff, which is then used to calculate an overall score (0 = worst situation, 100 = best situation).^24^ The ASCOT-Carer is a preference measure of social CRQoL. As with the CES and the CarerQoL-7D, the ASCOT-Carer is designed for self-completion by informal carers, although an interview version of this measure also exists. The measure contains 7 items, and relative weights attached to each item are summed to form a scale ranging from 0 (lowest) to 1 (highest social CRQoL).^25^ The EQ-5D-5L and ICECAP-A were included in the questionnaire as comparator measures that might also be used with carers. The EQ-5D-5L contains 5 items representing key aspects of HRQoL estimated on a 0 (death) to 1 (full health) scale.^34^ The ICECAP-A measure of capability well-being was developed for the general adult population and contains 5 attributes of capability well-being. Relative weights attached to each of these dimensions are aggregated to provide an overall score (0 = no capability to 1 = full capability).^26^ All measures were scored using UK population tariffs.^40^, ^41^, ^42^, ^43^, ^44^

**Table 1:** Conceptual mapping of domains in the Carer Experience Scale, CarerQoL, ASCOT-Carer, EQ-5D-5L, and ICECAP-A.

	Occupation	Support	Fulfillment	Control	Relationship	Social participation	Physical health	Mental health	Self-care	Safety and stability	Finances	Achievement
CES	Activities outside caring	Support from friends and family Assistance from organizations and government	Fulfillment from caring	Control over caring	Getting on with the person you care for	Included under “activities outside of caring”						
CarerQoL	Problems with combining care tasks with daily activities	Support with carrying out care tasks, as needed	Fulfillment from carrying out care tasks		Relational problems with the care receiver		Problems with physical health	Problems with mental health			Financial problems due to care tasks	
ASCOT-Carer	Occupation in valuable or enjoyable activities Space and time to be yourself	Feeling supported and encouraged		Control over daily life		Social contact with people you like			How well you look after yourself	How safe you feel		
EQ-5D-5L	Usual activities						Pain/discomfort Mobility	Anxiety/depression	Self-care			
ICECAP-A		Love, friendship, and support	Enjoyment and pleasure	Being independent						Feeling settled and secure		Achievement and progress

The baseline survey also contained contextual questions related to the carer, the care recipient (proxy reported by the carer), and the caring situation. These questions were chosen on the basis of evidence about factors associated with QoL from the literature. Table 2 provides further detail on the contextual questions in the baseline and follow-up surveys, along with evidence supporting the rationale for including the constructs and the hypothesized relationship with QoL scores.

**Table 2:** Contextual constructs captured in the baseline and follow-up surveys and their expected association with care-related QoL.

Contextual constructs∗	Included in:			Expected association with care-related QoL∗
	Baseline survey	Follow-up survey	Hypothesis development	
Related to the carer				
Age	✓	×	✓	Negative†
Gender	✓	×	✓	Negative
Ethnicity	✓	×	×	×
Educational qualification	✓	×	×	×
Occupation	✓	✓	✓	Positive‡
Impacts on occupation as a result of caring	✓	×	×	×
Self-rated life satisfaction	✓	✓	✓	Positive
Related to the care recipient				
Age	✓	×	✓	Positive§
Gender	✓	×	✓	Negative
Cognitive difficulties and daily dependencies	✓	✓	✓	Negative
Health status	✓	✓	✓	Positive
Anticipated direction of health status	✓	✓	✓	Negative
Related to the caring situation				
Co-residence	✓	✓	✓	Negative
Number of people who live with the carer	✓	✓	×	×
Relationship	✓	×	✓	Negative
Duration of caring	✓	×	✓	Negative§
Hours of care per week	✓	✓	✓	Negative
Time spent on specific care activities	✓	✓	✓	Negative
Perception of spillover effects from health and social care interventions	✓	×	×	×
Main carer	✓	×	✓	Negative
Involvement of others in the caring	✓	×	✓	Positive
Alternative ways they could spend their time	×	✓	×	×

QoL indicates quality of life.

∗ Key evidence for construct and hypothesis development.^3^^,^^20^^,^^22^^,^^35^^,^^38^^,^^47^, ^48^, ^49^, ^50^, ^51^, ^52^, ^53^

† Negative association expected for each condition with the exception of stroke (positive association expected).^45^

‡ Positive association expected for each condition with the exception of stroke and mental health (negative association expected).^22^^,^^46^

§ Negative association expected for each condition with the exception of stroke (positive association expected).

The survey questionnaire was piloted and developed through meetings with a lived experience advisory panel (LEAP). The 5 members of the LEAP brought lived experience in providing informal care. There was representation across disease areas and relationship to the care recipient. Panel members were recruited through lay groups attached to dementia, mental illness, and stroke charities. The baseline survey questionnaire was posted to the identified sample of carers (N = 1004) at the end of October 2016. Participants were asked to return the completed questionnaire within 3 weeks and were given 2 reminders.

If participants were not still providing care for the baseline care recipient, they were asked to complete the 2 generic QoL measures in a follow-up questionnaire 12 months later. Participants who were still in a caring role were asked to complete all QoL measures and contextual questions related to the carer, the care recipient, and the caring situation. Table 2 provides further detail on the contextual questions in the baseline and follow-up surveys, along with evidence supporting the rationale for including the constructs in the validity tests and the hypothesized relationship with QoL scores.

### Construct Validity Analysis

The construct validity of the measures was first assessed using convergent validation, which evaluates the extent to which one measure correlates with another measure of the same construct.^54^ It was expected that the 3 CRQoL measures would all be strongly correlated with each other. It was also hypothesized that the ICECAP-A score would be associated with the EQ-5D-5L score and that the CRQoL measures would be moderately correlated with the ICECAP-A and EQ-5D-5L measures. Convergent validity was analyzed using Spearman’s rank correlation coefficients to assess the strength, statistical significance, and direction of associations between scores of the different QoL measures.

Construct validity tests were based on prespecified evidence-based hypotheses about the way in which traits assumed to influence carer QoL would be linked to QoL measure scores.^55^ The main construct validity analysis examined traits positively and negatively associated with carers’ QoL. Two analyses were conducted to explore (1) the relationship between constructs (relating to patient characteristics and health difficulties, carer characteristics, and caring situation) and QoL measure scores for all conditions pooled together and (2) the relationship between constructs and QoL measure scores for each specified condition.

Hypotheses about how QoL measure scores were expected to relate to contextual constructs included in the survey were formed (see Table 2). Data from development and validation studies of the CES, CarerQoL-7D, ASCOT-Carer, EQ-5D-5L, and ICECAP-A measures were used to assist hypothesis formation (eg, see Goranitis et al,^27^ Rand et al,^25^ and Hoefman et al^29^). Existing evidence was supplemented with information from the broader literature^20^^,^^22^^,^^35^^,^^45^^,^^47^^,^^49^^,^^56^ on the caring experience and the views of the authors. Hypotheses regarding the impact of each construct on the QoL of carers were formed for all conditions pooled together. Separate hypotheses were also developed, where evidence was available, for the 4 conditions (see Table 2). Spearman rank correlation coefficients were computed to assess associations between QoL measure scores and constructs that were measured on a continuous scale (eg, carer age). Spearman rank correlation is a widely used correlation statistic to measure the degree of the relationship between continuous variables.^29^^,^^30^ Correlation values less than 0.3 were described as weak, values from 0.3 to 0.6 were described as moderate, and values of 0.6 and greater were described as strong.^57^ *t* Tests were used to test the significance of differences in QoL measure score between groups (eg, men vs women, spouse vs other relationship). Only participants who had completed both the QoL measure and constructs involved in a validity test were included in the analysis. Where carers reported multiple conditions for a patient (eg, dementia and stroke), the response was used for both subgroups.

### Responsiveness Analysis

An anchor-based approach was used to assess the relationship between the change in scores of a measure and changes in the scores of a related construct or measure (anchor).^58^ Care recipient HRQoL (as measured by the EQ-5D-5L) and informal care hours were selected as anchors based on their conceptual and empirical relationship with the informal carers’ QoL.^16^^,^^19^^,^^20^^,^^45^ The anchors were subdivided into 3 levels to indicate whether the anchor had increased, decreased, or not changed in an important way over time.^19^^,^^58^ A minimal clinically important difference of 0.063 for the EQ-5D-5L index score has been estimated.^59^ An “important” increase/decrease in care recipient EQ-5D-5L score was therefore defined as a minimal clinically important difference in scores between the 2 periods of at least 0.063. Literature on providing informal care categorizes an intensive level of caring as providing more than 20 or 50 hours of informal care per week.^60^^,^^61^ An “important” increase/decrease in the hours of care provided per week was therefore defined as a movement though a threshold of either 20 or 50 hours of care per week.

Responsiveness was evaluated using the standardized response mean effect size statistic, calculated as the ratio of the mean change between baseline and follow-up index scores to the standard deviation of the change scores.^62^ Assessments were made about the magnitude of response by calculating effect sizes for increases/decreases in QoL in the change groups using Cohen’s *d*. Hypotheses for responsiveness tests were consistent with the construct validity tests. An improvement in QoL was hypothesized in relation to a significant improvement in patient HRQoL (and vice versa for a worsening in patient HRQoL). An improvement in carer QoL was also hypothesized in relation to a significant reduction in caring hours (and vice versa for an increase in caring hours). For Cohen’s *d*, effect sizes between 0.2 and 0.5 are considered small, between 0.5 and 0.8 moderate, and >0.8 large.^63^ An assessment was also made of whether there was an expected gradient of effect in the QoL change scores,^19^ that is, whether the measured change in QoL over 12 months for the 3 subgroups of carers (denoted by the anchor categories) was ordered in the expected direction in relation to the change in the construct. Only individuals who had a complete set of item responses for the responsiveness tests were included in the analysis.

## Results

### Response Rate and Characteristics of Participants

The characteristics of participants are presented in Table 3. The overall response rate to the baseline questionnaire follow-up reminders was 57% (n = 576), and these participants form the basis of the construct validity analysis. Of the 576 respondents, 65% were female. The age of participants ranged from 24 to 89 years, with a mean age of 62 years (SD = 11). Just less than half of participants (46%) were caring for a parent and 35% for their spouse/partner. Further characteristics of participants categorized by condition are presented in Appendix 1 in the Supplemental Materials found at https://doi.org/10.1016/j.jval.2020.01.015. The follow-up questionnaire was posted to the 576 participants who responded at baseline. In total, 431 (75%) responses were received with 117 (27%) not eligible because the participant no longer provided care (n = 89) or was providing care for a different person (n = 28), leaving 314 (73%) eligible responses. These individuals were still providing informal care to the person they were caring for at baseline and form the basis of the responsiveness analysis.

**Table 3:** Characteristics of the carer, care recipient, and caring situation (all conditions) for the sample of carers included in the construct validity and responsiveness analysis.

Construct	Construct validity sample at baseline (n = 576)∗	Responsiveness sample at baseline (n = 314)∗	Responsiveness sample at follow-up (n = 314)∗
Carer			
Age, y, mean (SD)	62 (11.10)	63 (10.01)	64 (10.01)†
Gender, female, n (%)	367 (64)	191 (63)	191 (63)†
Health status (EQ-5D-5L), mean (SD)	0.76 (0.21)	0.80 (0.21)	0.74 (0.23)
Occupation, in paid employment, n (%)	189 (33)	84 (14)	84 (14)
Self-rated life satisfaction, scale 0-10, mean (SD)	6.7 (2.23)	6.7 (2.33)	7 (2.24)
Care recipient			
Age, y, mean (SD)	74 (18.33)	73 (19.11)	74 (19.11)†
Gender, male, n (%)	203 (35)	108 (36)	108 (36)†
Has cognitive problems, yes, n (%)	340 (59)	177 (58)	205 (68)
Has daily dependencies, yes, n (%)	518 (90)	269 (89)	279 (93)
Health status (EQ-5D-5L), mean (SD)	0.32 (0.31)	0.30 (0.35)	0.31 (0.35)
Direction of health status, declining, n (%)	373 (66)	191 (63)	174 (58)
Caring situation			
Co-residence, yes, n (%)	266 (46)	153 (51)	156 (52)
Relationship to carer, spouse, n (%)	199 (35)	111 (37)	111 (37)†
Duration of caring, years, mean (SD)	9.9 (10.20)	10.9 (126.5)	11.9 (126.5)†
Time spent caring >20 h per wk, n (%)	295 (51)	183 (60)	193 (64)
Time spent caring >50 h per wk, n (%)	165 (29)	100 (32)	115 (37)
Provides personal care, n (%)	343 (68)	183 (60)	218 (72)
Identifies as the main carer, yes, n (%)	408 (72)	233 (77)	233 (77)†
Other people are involved in the caring, yes, n (%)	367 (65)	182 (61)	182 (61)†

∗ Pairwise associations are based on complete cases in which participants completed both the quality-of-life measure and the constructs involved in the validity test.

† Question not asked in the follow-up questionnaire.

The response rate for QoL measures varied between 89% and 98% at baseline and between 96% and 98% at follow-up. Among the CRQoL measures, the ASCOT-Carer had the highest response rate at baseline (96%) and follow-up (98%). The mean ASCOT-Carer score was 0.74 at baseline and follow-up. There was a slight rise in the mean score in the CES (62.9 to 65.1) and CarerQoL-7D (72.3 to 73.7) in the 12-month period from baseline to follow-up. The mean EQ-5D-5L score for carers decreased from 0.79 to 0.74, whereas the mean score for the ICECAP-A increased from 0.76 to 0.81 at follow-up. Further detail on the response rate for the measures and the change in mean scores over time are provided in Appendix 2 of the Supplemental Materials found at https://doi.org/10.1016/j.jval.2020.01.015.

### Construct Validity Analysis

#### Convergent validation

As expected, the CRQoL measures were all strongly correlated with each other (Table 4). The ICECAP-A was also strongly correlated with the CRQoL measures, with some correlations stronger than those found between the CRQoL measures themselves. The EQ-5D-5L was not strongly correlated with any of the other measures and was weakly correlated with the CES (correlation coefficient 0.25).

**Table 4:** Correlations between CES, CarerQoL, ASCOT-Carer, EQ-5D-5L, and ICECAP-A measures index scores.

QoL measure	CES	CarerQoL	ASCOT-Carer	EQ-5D-5L (carer)	ICECAP-A
CES					
CarerQoL	0.56∗				
ASCOT-Carer	0.60∗	0.71∗			
EQ-5D-5L (carer)	0.25∗	0.51∗	0.44∗		
ICECAP-A	0.57∗	0.69∗	0.85∗	0.50∗	

∗ *P* < .001.

#### Association between the overall measure scores for all conditions and contextual constructs

Table 5 shows the associations and effect sizes between contextual constructs and QoL measure score. A statistically significant association (in the expected direction) was detected in each of the 5 measures in 14 of 16 tests. Larger effect sizes and stronger associations were generally detected between the contextual constructs and the ASCOT-Carer and ICECAP-A measures. Focusing on constructs relating to patient health (symptoms and EQ-5D-5L score), the moderate or large effects were detected for the CarerQoL in addition to the ASCOT-carer and ICECAP-A. Focusing on constructs related to the caring situation (hours of care, provision of personal care, main carer role), moderate to large effects were detected for the ASCOT-carer and ICECAP-A.

**Table 5:** Univariable associations and effect sizes between quality-of-life measure scores (all conditions) and contextual constructs.

Contextual construct		CES		CarerQoL		ASCOT-Carer		EQ-5D-5L (carer)		ICECAP-A	
	n	CC	Effect size	CC	Effect size	CC	Effect size	CC	Effect size	CC	Effect size
Carer											
Age	558	−0.02		0.06		−0.01		−0.15∗∗∗		0.01	
Gender, female	569		−0.18∗		−0.23∗∗		−0.16		−0.01		−0.06
Employment status, paid employment	567		0.25∗∗		0.23∗∗		0.17		0.38∗∗∗		0.22∗∗
Self-rated life satisfaction	564	0.52∗∗∗		0.64∗∗∗		0.78∗∗∗		0.43∗∗∗		0.82∗∗∗	
Care recipient											
Age	546	0.10∗		0.15∗∗		0.17∗∗∗		0.14∗∗		0.23∗∗∗	
Gender, male	574		−0.06		−0.27∗∗		−0.22∗		−0.19∗		−0.30∗∗∗
Cognitive difficulties and daily dependencies	576	−0.14∗∗∗		−0.35∗∗∗		−0.39∗∗∗		−0.18∗∗∗		−0.38∗∗∗	
Health status (EQ-5D-5L)	503	0.15∗∗∗		0.30∗∗∗		0.32∗∗∗		0.24∗∗∗		0.32∗∗∗	
Direction of health status, declining	569		−0.34∗∗∗		−0.34∗∗∗		−0.29∗∗		−0.17∗		−0.24∗∗
Caring situation											
Co-residence	575		−0.49∗∗∗		−0.33∗∗∗		−0.62∗∗∗		−0.40∗∗∗		−0.63∗∗∗
Relationship, spouse	576		−0.21∗		−0.16		−0.35∗∗∗		−0.37∗∗∗		−0.46∗∗∗
Duration of caring (months)	568	−0.10∗		−0.14∗∗∗		−0.17∗∗∗		−0.12∗∗		−0.21∗∗∗	
Hours of care per week >20	559		−0.58∗∗∗		−0.62∗∗		0.94∗∗∗		−0.45∗∗∗		−0.86∗∗∗
Provides personal care	508		−0.31∗∗		−0.47∗∗∗		−0.64∗∗∗		−0.42∗∗∗		−0.61∗∗
Main carer	566		−0.59∗∗∗		−0.49∗∗∗		−0.62∗∗∗		−0.34∗∗∗		−0.57∗∗∗
Involvement of others	566		0.44∗∗∗		0.21∗		0.22∗		0.13		0.22∗∗

*Note.* CC indicates correlation coefficient. Spearman’s rho is reported for all constructs that are continuous variables (age, self-rated life satisfaction, number of cognitive difficulties and daily dependencies, recipient EQ-5D-5L, duration of caring). Cohen’s *d* effect size is reported for all other variables. Spearman’s rank correlation coefficients of >0.3 are considered weak, >0.5 moderate, and >0.7 strong. Cohen’s *d* effect sizes of >0.2 are considered small, >0.5 moderate, and >0.8 large. The same interpretations apply for negative correlation coefficients and effect sizes. Yellow shading indicates not statistically significant; light yellow shading, statistically significant, small/weak effect size; dark yellow shading, statistically significant, moderate/high/strong effect size.

∗ *P*<.05;

∗∗ *P*<.01;

∗∗∗ *P*<.001.

#### Association between the overall measure scores for the different conditions and contextual constructs

The results for the tests of construct validity for dementia, stroke, mental illness, and rheumatoid arthritis are presented in Table 6, with results summarized to indicate the number of significant associations at the 5% level between the group of constructs (eg, carer constructs for dementia carers) and the measure scores identified. Overall, of the 3 CRQoL measures, the ASCOT-Carer detected more statistically significant associations at the 5% level for each condition than the CES or CarerQoL-7D. Of the 2 other measures, the ICECAP-A performed slightly better than the EQ-5D-5L for each condition and particularly for caring situation constructs for stroke. All hypothesized associations were in the expected direction. For dementia, the CarerQoL-7D and ASCOT-Carer had the most significant associations (12 of 27) among care-related measures—equal with the better-performing comparator measure (ICECAP-A). For stroke, of the CRQoL measures, the ASCOT-Carer had the most significant associations (18 of 27), 1 more than the better-performing comparator measure (ICECAP-A). The ASCOT-Carer, along with the CarerQoL-7D, had the most significant associations for mental illness (10 of 27), and this was equal to both the ICECAP-A and the EQ-5D-5L. Finally, for rheumatoid arthritis, the ICECAP-A had the most significant associations (11 of 27), 1 more than the 3 CRQoL measures and the EQ-5D-5L.

**Table 6:** Frequency of statistically significant associations detected between QoL measure scores and contextual constructs.

Condition	Category	CES	CarerQoL	ASCOT-Carer	EQ-5D-5L (carer)	ICECAP-A
Dementia (n = 155)	Carer∗	2/5	3/5	2/5	2/4	2/5
	Care recipient	0/5	2/5	2/5	2/5	2/5
	Caring situation	4/7	4/7	6/7	5/7	6/7
	Health difficulties†	2/10	3/10	2/10	1/10	2/10
Stroke (n = 89)	Carer∗	2/5	3/5	2/5	1/4	2/5
	Care recipient	1/5	2/5	3/5	2/5	3/5
	Caring situation	6/7	3/7	6/7	2/7	6/7
	Health difficulties†	3/10	5/10	7/10	3/10	6/10
Mental illness (n = 144)	Carer∗	2/5	2/5	2/5	2/4	2/5
	Care recipient	1/5	4/5	3/5	3/5	4/5
	Caring situation	1/7	0/7	3/7	3/7	2/7
	Health difficulties†	0/10	4/10	2/10	2/10	2/10
Rheumatoid arthritis (n = 126)	Carer∗	3/5	2/5	2/5	2/4	2/5
	Care recipient	2/5	3/5	3/5	2/5	3/5
	Caring situation	4/7	4/7	5/7	4/7	5/7
	Health difficulties†	1/8	1/8	0/8	2/8	1/8

QoL indicates quality of life.

∗ One of the contextual constructs under the carer category is self-reported health as measured by the EQ-5D-5L. The relationship between EQ-5D-5L (carer) score and QoL measure score was analyzed for the CES, CarerQoL, ASCOT-Carer, and ICECAP-A, resulting in 5 contextual constructs for these measures and 4 constructs for the EQ-5D-5L (carer).

† A full list of the health difficulties included for analysis is provided in Appendix 3 of the Supplemental Materials. It was anticipated that the presence of any of these difficulties would have a negative impact on care-related QoL.

### Responsiveness Analysis

#### Anchor of change: Care recipient health status

Overall, 94 participants reported that the care recipient’s HRQoL had improved at follow-up, 80 reported no change, and 97 participants reported that the care recipient’s HRQoL was worse at follow-up. As detailed in Table 7, of the CRQoL measures the CarerQoL-7D appears to be more responsive than the CES and ASCOT-Carer. Larger effect sizes were detected for the CES, but a slight gradient of effect in line with care recipient HRQoL was detected for the CarerQoL-7D, although the effect sizes were trivial/small. Similar effect sizes were detected for the 2 comparator QoL measures as care recipient HRQoL improved or worsened, although these effects were in contrasting directions.

**Table 7:** Responsiveness of QoL measures by care recipient health status and hours of care provided per week.

	n	Baseline score, mean (SD)	Follow-up score, mean (SD)	Score change, mean (SD)	SRM	Effect size
Care recipient health status (meaningful change at 0.063)						
CES						
Improved	81	62.14 (17.41)	66.03 (18.97)	3.88 (17.67)	0.22	Small
No change	73	65.18 (19.05)	64.24 (19.51)	−0.94 (13.68)	−0.07	
Worsened	83	61.77 (19.15)	66.11 (19.06)	4.34 (20.44)	0.21	Small
CarerQoL						
Improved	82	72.75 (21.03)	75.68 (18.11)	2.94 (17.55)	0.17	
No change	68	72.60 (22.13)	72.99 (21.27)	0.39 (13.80)	0.03	
Worsened	82	70.99 (19.91)	71.06 (20.90)	0.07 (15.39)	0.00	
ASCOT-carer						
Improved	91	0.74 (0.21)	0.76 (0.21)	0.02 (0.15)	0.10	
No change	75	0.77 (0.22)	0.77 (0.21)	0.00 (0.12)	0.03	
Worsened	91	0.74 (0.21)	0.76 (0.18)	0.02 (0.18)	0.08	
EQ-5D-5L						
Improved	92	0.80 (0.21)	0.74 (0.20)	−0.05 (0.20)	−0.26	Small
No change	79	0.80 (0.20)	0.76 (0.22)	−0.04 (0.14)	−0.28	Small
Worsened	93	0.80 (0.22)	0.72 (0.22)	−0.09 (0.16)	−0.54	Moderate
ICECAP-A						
Improved	91	0.75 (0.21)	0.81 (0.17)	0.05 (0.16)	0.31	Small
No change	76	0.76 (0.22)	0.83 (0.16)	0.07 (0.17)	0.40	Small
Worsened	92	0.77 (0.21)	0.81 (0.18)	0.04 (0.18)	0.21	Small
Hours of care provided per week (threshold of ±20 h and ±50 h of care per week)						
CES						
Less hours	44	59.34 (20.69)	64.91 (21.05)	5.38 (22.57)	0.24	Small
No change	177	62.95(18.49)	65.02 (19.39)	1.53 (16.67)	0.09	
More hours	45	64.95 (18.17)	65.61 (18.69)	0.57 (15.70)	0.04	
CarerQoL						
Less hours	43	68.68 (23.28)	74.06 (18.82)	6.02 (21.13)	0.28	Small
No change	168	72.33 (21.56)	73.44 (21.74)	1.30 (14.54)	0.09	
More hours	48	74.69 (20.84)	74.15 (18.79)	−0.06 (13.50)	0.00	
ASCOT-Carer						
Less hours	45	0.70 (0.21)	0.75 (0.19)	0.05 (0.19)	0.26	Small
No change	189	0.74 (0.23)	0.74 (0.23)	0.01 (0.14)	0.07	
More hours	56	0.78 (0.20)	0.75 (0.19)	−0.03 (0.13)	−0.25	Small
EQ-5D-5L						
Less hours	48	0.77 (0.23)	0.74 (0.18)	−0.04 (0.20)	−0.20	Small
No change	190	0.80 (0.21)	0.74 (0.25)	−0.06 (0.17)	−0.36	Small
More hours	55	0.82 (0.19)	0.73 (0.19)	−0.09 (0.20)	−0.45	Small
ICECAP-A						
Less hours	45	0.74 (0.21)	0.83 (0.14)	0.08 (0.21)	0.40	Small
No change	184	0.75 (0.22)	0.81 (0.19)	0.06 (0.15)	0.36	Small
More hours	57	0.80 (0.19)	0.78 (0.19)	−0.02 (0.19)	−0.11	

QoL indicates quality of life; SRM, standardized response mean.

#### Anchor of change: Hours of care per week

Using the threshold of 20 hours and 50 hours per week 49 participants reported in the follow-up questionnaire that they were providing less hours of care per week, 193 reported no change, and 61 participants reported that they were providing more hours of care at follow up. A slight gradient of effect in QoL change scores was found in relation to the number of hours of care provided per week for each of the 3 CRQoL measures. The change in QoL score was larger for the ASCOT-Carer compared with the CES and CarerQoL-7D, although effect sizes ranged from trivial to small for each measure. A gradient of effect as caring hours moved from a decrease to an increase was found for the ICECAP-A and the EQ-5D-5L scores.

## Discussion

This study investigates, for the first time, the psychometric performance of different preference-based measures of carer QoL for different groups of informal carers. The study focused on the performance of 3 care-related QoL measures, the CES, CarerQoL-7D, ASCOT-Carer; 1 health-related QoL measure, the EQ-5D-5L; and 1 generic QoL measure, the ICECAP-A, for capturing and measuring carer effects for use in economic evaluation. The construct validity and responsiveness of each measure were investigated in informal carers of adults with dementia, stroke, mental illness, or rheumatoid arthritis. The findings from the results suggest that each measure exhibits some degree of psychometric validity in measuring QoL for carers.

In terms of construct validity, across all conditions, of the 3 CRQoL measures, more statistically significant associations were found in relation to the ASCOT-Carer compared with the CES or the CarerQoL-7D. Of the other measures, the ICECAP-A exhibited greater construct validity than the EQ-5D-5L. The ASCOT-Carer and ICECAP-A were also comparable in the sense that larger effect sizes and stronger associations were detected for these measures, relative to the other measures, when the conditions were analyzed separately.

No measure exhibited clear responsiveness to changes within a year in care recipient health status or hours of care provided per week. Of the CRQoL measures, the CarerQoL-7D detected a slight gradient of effect, suggesting that it may be more responsive than the CES and the ASCOT-Carer, although effect sizes were small. These results need to be viewed in the context of the changing access to services and distribution of care tasks and changes in underlying level of QoL reported by the carers in the sample. In particular, there was an underlying increase in capability well-being and decrease in HRQoL in the carer population. This is feasible given the limited conceptual overlap between the EQ-5D-5L and ICECAP-A,^64^ as detailed in Table 1. It may also be explained by the ICEAP-A attributes of control and fulfilment, for which the largest improvements in overall capability over 12 months were reported, and the pain/discomfort attribute and mental health attribute, for which the largest decline in HRQoL was seen. Given the underlying fluctuations in HRQoL and capability well-being over 12 months, the gradient of effect results may provide a more meaningful indication of responsiveness (or at least sensitivity) of the QoL measures than the standardized response mean or effect size.^19^

The construct validity findings for each measure were in line with the available literature in the field in the sense that many of the constructs were supported, particularly in the pooled analysis.^25^^,^^27^^,^^29^^,^^30^^,^^33^^,^^65^ The findings also complement existing validity literature that indicates that the ASCOT and ICECAP measures may be more sensitive than the EQ-5D in studies in which the main objective of an intervention is broader than just improving or maintaining health.^66^

The performance of the ICECAP-A and ASCOT-Carer reinforce the idea that focusing on outcomes broader than health for carers is more appropriate and provides encouragement in using CRQoL and capability in economic evaluation. What was perhaps unexpected is that the results show that CRQoL measures are not always more sensitive to constructs hypothesized to be associated with QoL of carers. In terms of construct validity, the ICECAP-A emerged as being comparable with the best-performing care-related measure, the ASCOT-Carer. The ICECAP-A attributes are predominantly psychosocial,^64^ and this could explain its comparative sensitivity to constructs associated with carer QoL.

This study has several implications for economic evaluation and health technology assessment. If the focus is on health maximization, this study suggests that the EQ-5D-5L has relatively encouraging validity as an outcome measure with informal carers. If there is more flexibility in the economic evaluation, the findings suggest that the ICECAP-A and measures of CRQoL (in particular, the ASCOT-Carer) may be attractive to include in addition or instead of an HRQoL measure, in view of their performance. If capability or CRQoL measures are included in addition to an HRQoL measure for carers, care would need to be taken to avoid double counting in the presentation of the results.

When interpreting the results of this study, some limitations have to be considered. First, there were relatively small subsamples of carers for the individual conditions analyzed. This reduced the power to detect associations between the overall measure scores and contextual constructs. This was also the case for the responsiveness analysis. Second, the sample of carers for this study was drawn across the (then) 3 most recent waves of the FRS (2013-2016). This sampling method was not conducive to identifying carers new to the caring role. The sample of carers in this study had been providing care for an average of 9 years. This limitation needs to be considered alongside the difficulties in accessing carers for research and this approach did enable access to a relatively large sample of family carers across the United Kingdom, with experiences of caring across the 4 major conditions. Third, only 57% of carers completed the 12-month follow-up questionnaire. Although this reflects the frequent transitions in and out of caring role, it does mean the sample for the responsiveness analysis is smaller and has a different composition from the construct validity sample. Fourth, family carers proxy reported details about the patients’ health and symptoms. This was done for practicality reasons, given a large (unknown) number of patients would be unable to self-report their health. A previous study found no evidence that this form of proxy reporting leads to artificially high correlation rates between patient and carer outcomes.^2^ Nevertheless, studies have suggested that proxy reports do not always match self-reports.^67^

The results of this study provide encouraging initial evidence of the validity and mixed evidence of responsiveness of care-related and generic QoL measures in informal carers of adults suffering from 4 highly prevalent conditions associated with diverse impacts on carers’ lives. The mixed evidence on responsiveness may in part be due to the nature of the sample, and this is an area for further research. Nevertheless, a tentative conclusion emerges that the generic measure of QoL, the ICECAP-A, performs as well as CRQoL measures and better than the HRQoL measure in detecting impacts of caring on QoL.

## Supplemental Material

Appendices 1-3

Instruments
