# Supplementary material for: Validity and Responsiveness of Preference-Based Quality-of-Life Measures in Informal Carers: A Comparison of 5 Measures Across 4 Conditions
Source: Value Health. 2020 Jun;23(6):782–90. doi: 10.1016/j.jval.2020.01.015 (PMC7532692; doi:10.1016/j.jval.2020.01.015)
Supplement: Appendices 1-3 [file mmc1.docx]

Appendix 1 Characteristics of the carer, care recipient and caring situation for individual conditions for the sample of carers included in the construct validity analysis

| **Construct** | **Dementia**  **(n=155)** | | **Stroke**  **(n=89)** | **Mental illness**  **(n=144)** | **Rheumatoid arthritis**  **(n=126)** |
| --- | --- | --- | --- | --- | --- |
| **Carer** | | | | | |
| Age, years, mean (SD) | 64 (11.32) | 64 (10.70) | | 61 (11.53) | 61 (12.27) |
| Gender, female, n (%) | 107 (69) | 61 (69) | | 95 (66) | 71 (56) |
| Health status (EQ-5D-5L), mean (SD) | 0.77 (0.22) | 0.74 (0.21) | | 0.69 (0.25) | 0.74 (0.21) |
| Occupation, in paid employment, n (%) | 43 (28) | 30 (34) | | 45 (31) | 42 (34) |
| Self-rated life satisfaction, scale 0-10, mean (SD) | 6.5 (2.12) | 6.7 (2.36) | | 6.2 (2.33) | 7.1 (2.34) |
| **Care recipient** | | | | | |
| Age, years, mean (SD) | 84 (7.88) | 77 (13.66) | | 65 (20.18) | 80 (13.03) |
| Gender, male, n (%) | 48 (31) | 37 (42) | | 49 (34) | 32 (25) |
| Has cognitive problems, yes, n (%) | 151 (97) | 66 (74) | | 99 (69) | 59 (47) |
| Has daily dependencies, yes, n (%) | 142 (92) | 82 (92) | | 135 (94) | 113 (90) |
| Health status (EQ-5D-5L), mean (SD) | 0.31 (0.32) | 0.32 (0.33) | | 0.26 (0.32) | 0.24 (0.29) |
| Direction of health status, declining, n (%) | 127 (83) | 58 (65) | | 86 (60) | 97 (78) |
| **Caring situation** | | | | | |
| Co-residence, yes, n (%) | 55 (36) | 43 (48) | | 75 (52) | 50 (40) |
| Relationship to carer, spouse, n (%) | 43 (27) | 33 (37) | | 54 (38) | 39 (31) |
| Duration of caring, years, mean (SD) | 6.4 (5.91) | 10.2 (10.2) | | 13.1 (10.9) | 7.6 (8.11) |
| Time spent caring >20 hours per week, n (%) | 66 (44) | 53 (60) | | 95 (66) | 60 (49) |
| Provides personal care, n (%) | 81 (62) | 62 (76) | | 100 (75) | 73 (63) |
| Identifies as the main carer, yes, n (%) | 99 (66) | 61 (70) | | 117 (82) | 92 (74) |
| Other people are involved in the caring, yes, n (%) | 120 (79) | 65 (74) | | 83 (58) | 85 (69) |

Appendix 2 Quality of life measures response rate and mean score at baseline and follow up

| **Measure** | **Sample at baseline (n=576)** | | **Sample at follow up (n=314)** | |
| --- | --- | --- | --- | --- |
|  | **Response rate %** | **Mean score** | **Response rate %** | **Mean score** |
| Carer Experience Scale | 89 | 62.9 | 96 | 65.1 |
| CarerQoL | 89 | 72.3 | 96 | 73.7 |
| ASCOT-Carer | 96 | 13.8 | 98 | 14.2 |
| EQ-5D-5L (carer) | 98 | 0.79 | 98 | 0.74 |
| EQ-5D-5L (care recipient) | 98 | 0.30 | 96 | 0.31 |
| ICECAP-A | 94 | 0.76 | 98 | 0.81 |

**Appendix 3 Condition specific health difficulties associated with dementia, stroke, mental health condition, rheumatoid arthritis**

| Dementia [[65](#_ENREF_65)] | Stroke [[66](#_ENREF_66)] | Mental Health Condition [[67](#_ENREF_67)] | Rheumatoid Arthritis [[68](#_ENREF_68)] |
| --- | --- | --- | --- |
| *Mislaying or hiding items around the house* | Problems with co-ordination and balance | *Delusions, paranoia or hallucinations* | Sleep disturbance / difficulties |
| *Forgetting recent conversations or events* | Sleep disturbance / difficulties | *Confused or racing thoughts* | *Pain and stiffness of affected joints* |
| Struggle to find the right word or keep track of conversation | *Changes to emotions e.g. feelings of misery or anxiety* | *Diminished emotional expression* | *Loss of appetite or weight loss* |
| Grasping new ideas and unwilling to try out new things | Problems with swallowing | Inappropriate or unpredictable behaviour | Swelling of affected joints, joints become hot and tender to touch |
| *Losing track of the day or date* | *Visual problems e.g. double vision, reduced field of vision* | *Emotional withdrawal* | *Pinched or compressed nerves e.g. carpal tunnel syndrome* |
| Withdrawal from friends and activities | Irritability or rudeness | Changes in sleeping patterns | Skin complications e.g. rash, ulcers, blisters, lumps under the skin |
| Showing confusion about where they are or walking off | Communication problems i.e. difficulty with speech and language | Feelings of helplessness and hopelessness | Changes to emotions e.g. feelings of misery or anxiety |
| Unusual behaviour (e.g. agitation in social situations) | Overdependence | Compulsive behaviour | *Struggle to carry out a familiar task (e.g. make a cup of tea)* |
| Delusions, paranoia or hallucinations | Muscle weakness or stiffness | Loss of energy |  |
| *Struggle to carry out a familiar task (e.g. make a cup of tea)* | Struggle to carry out a familiar task (e.g. make a cup of tea) | *Reckless behaviour (e.g. substance abuse or gambling)* |  |

Note: No statistically significant association was detected between health difficulties in italics and QoL measure scores for the CES, CarerQoL, ASCOT-Carer, EQ-5D-5L (carer), or ICECAP-A
