# Supplementary material for: Validity and Responsiveness of Preference-Based Quality-of-Life Measures in Informal Carers: A Comparison of 5 Measures Across 4 Conditions
Source: Value Health. 2020 Jun;23(6):782–90. doi: 10.1016/j.jval.2020.01.015 (PMC7532692; doi:10.1016/j.jval.2020.01.015)
Supplement: Instruments [file mmc2.doc]

**PREFERENCE BASED QUALITY OF LIFE MEASURES USED IN THIS STUDY**

1. **EQ-5D-5L**

**Under each heading, please tick the ONE box that best describes YOUR health today.**

**MOBILITY**

I have no problems in walking about _1_

I have slight problems in walking about _2_

I have moderate problems in walking about _3_

I have severe problems in walking about _4_

I am unable to walk about _5_

**SELF CARE**

I have no problems in washing and dressing myself _1_

I have slight problems in washing and dressing myself _2_

I have moderate problems in washing and dressing myself _3_

I have severe problems in washing and dressing myself _4_

I am unable to wash and dress myself _5_

**USUAL ACTIVITIES (e.g. work, study, housework, family or leisure)**

I have no problems doing my usual activities _1_

I have slight problems doing my usual activities _2_

I have moderate problems doing my usual activities _3_

I have severe problems doing my usual activities _4_

I am unable to do my usual activities _5_

**PAIN/DISCOMFORT**

I have no pain or discomfort _1_

I have slight pain or discomfort _2_

I have moderate pain or discomfort _3_

I have severe pain or discomfort _4_

I have extreme pain or discomfort _5_

**ANXIETY/DEPRESSION**

I have no anxiety or depression _1_

I have slight anxiety or depression _2_

I have moderate anxiety or depression _3_

I have severe anxiety or depression _4_

I have extreme anxiety or depression _5_

1. **ICECAP-A CAPABILITY MEASURE**

**Please indicate which statements best describe your overall quality of life at the moment by placing a tick in ONE box for each of the five aspects of quality of life below.**

**Feeling settled and secure**

I am able to feel settled and secure in **all** areas of my life _4_

I am able to feel settled and secure in **many** areas of my life _3_

I am able to feel settled and secure in **a few** areas of my life _2_

I am **unable** to feel settled and secure in **any** areas of my life _1_

**Love, friendship and support**

I can have **a lot** of love, friendship and support _4_

I can have **quite a lot** of love, friendship and support _3_

I can have **a little** love, friendship and support _2_

I **cannot** have **any** love, friendship and support _1_

**Being independent**

I am able to be **completely** independent _4_

I am able to be independent in **many** things _3_

I am able to be independent in **a few** things _2_

I am **unable** to be at all independent _1_

**Achievement and progress**

I can achieve and progress in **all** aspects of my life _4_

I can achieve and progress in **many** aspects of my life _3_

I can achieve and progress in **a few** aspects of my life _2_

I **cannot** achieve and progress in **any** aspects of my life _1_

**Enjoyment and pleasure**

I can have **a lot** of enjoyment and pleasure _4_

I can have **quite a lot** of enjoyment and pleasure _3_

I can have **a little** enjoyment and pleasure _2_

I **cannot** have **any** enjoyment and pleasure _1_

1. **CARERQOL MEASURE**

**Thinking about your current experience of caring for this person, please tick one box for each group to indicate which statement best describes your current caring situation.**

|  |  | **No** |  | **Some** |  | **A lot of** |  |
| --- | --- | --- | --- | --- | --- | --- | --- |
|  | I have | 1 |  | 2 |  | 3 | fulfillment with carrying out my care tasks |
|  | I have | 1 |  | 2 |  | 3 | relational problems with the care receiver *(e.g., he/she is very demanding; he/she behaves differently, we have communication problems)* |
|  | I have | 1 |  | 2 |  | 3 | problems with my own mental health *(e.g., stress, fear,* *gloominess, depression, concern about the future)*. |
|  | I have | 1 |  | 2 |  | 3 | problems combining my care tasks with my daily activities *(e.g., household activities, work, study, family and leisure activities).* |
|  | I have | 1 |  | 2 |  | 3 | financial problems because of my care tasks. |
|  | I have | 1 |  | 2 |  | 3 | support with carrying out my care tasks, when I need it *(e.g., from family, friends, neighbors, acquaintances)* |
|  | I have | 1 |  | 2 |  | 3 | problems with my own physical health *(e.g., more often sick, tiredness, physical stress)*. |

1. **CARER EXPERIENCE SCALE**

**Again, thinking about your current experience of caring for this person, please tick one box for each group to indicate which statement best describes your current caring situation.**

**Activities outside caring** *(Socialising, physical activity and spending time on hobbies, leisure or study)*

You can do most of the other things you want to do outside caring _1_

You can do some of the other things you want to do outside caring _2_

You can do few of the other things you want to do outside caring _3_

**SUPPORT FROM FAMILY AND FRIENDS** *(Personal help in caring and/or emotional support from family, friends, neighbours or work colleagues)*

You get a lot of support from family and friends _1_

You get some support from family and friends _2_

You get little support from family and friends _3_

**ASSISTANCE FROM ORGANISATIONS AND THE GOVERNMENT** *(Help from public, private or voluntary groups in terms of benefits, respite and practical information)*

You get a lot of assistance from organisations and the government _1_

You get some assistance from organisations and the government _2_

You get little assistance from organisations and the government _3_

**FULFILMENT FROM CARING** *(Positive feelings from providing care, which may come from: making the person you care for happy, maintaining their dignity, being appreciated, fulfilling your responsibility, gaining new skills or contributing to the care of the person you look after)*

You mostly find caring fulfilling _1_

You sometimes find caring fulfilling _2_

You rarely find caring fulfilling _3_

**CONTROL OVER THE CARING** *(Your ability to influence the overall care of the person you look after)*

You are in control of most aspects of the caring _1_

You are in control of some aspects of the caring _2_

You are in control of few aspects of the caring _3_

**GETTING ON WITH THE PERSON YOU CARE FOR** *(Being able to talk with the person you look after, and discuss things without arguing)*

You mostly get on with the person you care for _1_

You sometimes get on with the person you care for _2_

You rarely get on with the person you care for _3_

1. **ASCOT-CARER MEASURE**

**Finally, thinking about your current experience of caring for this person, please tick one box for each group to indicate which statement best describes your current situation.**

**Which of the following statements best describes how you spend your time?**

*When you are thinking about how you spend your time, please include anything you value or enjoy, including leisure activities, formal employment, voluntary or unpaid work, and caring for others.*

I’m able to spend my time as I want, doing things I value or enjoy _1_

I’m able to do enough of the things I value or enjoy with my time _2_

I do some of the things I value or enjoy with my time _3_

I don’t do anything I value or enjoy with my time _4_

**Which of the following statements best describes how much control you have over your daily life?**

I have as much control over my daily life as I want _1_

I have adequate control over my daily life _2_

I have some control over my daily life, but not enough _3_

I have no control over my daily life _4_

**Thinking about how well you look after yourself – such as, getting enough sleep or eating well – which statement best describes your present situation?**

I look after myself as well as I want _1_

I look after myself well enough _2_

Sometimes I can’t look after myself well enough _3_

I feel I am neglecting myself _4_

**Which of the following statements best describes how safe you feel?**

*By ‘feeling safe’ we mean feeling safe from fear of abuse, being attacked or other physical harm, such as accidents, which are a result of your caring role.*

I feel as safe as I want _1_

Generally I feel adequately safe, but not as safe as I would like _2_

I feel less than adequately safe _3_

I don’t feel at all safe _4_

**Thinking about how much contact you have with people you like, which of the following statements best describes your social situation?**

I have as much social contact as I want with people I like _1_

I have adequate social contact with people _2_

I have some social contact with people, but not enough _3_

I have little social contact with people and feel socially isolated _4_

**Thinking about the space and time you have to be yourself in your daily life, which of the following statements best describes your present situation?**

I have all the space and time I need to be myself _1_

I have adequate space and time to be myself _2_

I have some of the space and time I need to be myself, but not enough _3_

I don’t have any space or time to be myself _4_

**Thinking about feeling supported and encouraged in your caring role, which of the following statement best describes your present situation?**

*This question is asking about* ***feeling*** *supported and encouraged, rather than how you are supported and encouraged by particular people or organisations.*

I feel I have the encouragement and support I want _1_

I feel I have adequate encouragement and support _2_

I feel I have some encouragement and support, but not enough _3_

I feel I have no encouragement and support _4_
